# Gravity is vertical in Geophysical Fluid Dynamics

**DOI:** 10.1038/s41598-022-10023-3

**Published:** 2022-04-11

**Authors:** Andrew L. Stewart, James C. McWilliams

**Affiliations:** Department of Atmospheric and Oceanic Sciences, University of California, Los Angeles, CA USA

**Keywords:** Physical oceanography, Ocean sciences

**arising from**: P. C. Chu; *Scientific Reports* 10.1038/s41598-021-82882-1 (2021).

## Introduction

Earth is a rotating, gravitating planet, with space-time variations in these properties that are small compared to their average values. In analyzing Earth’s atmospheric and oceanic dynamics, it is often assumed that the gravitational acceleration *g* and the rotation rate ${\varvec{\Omega }}$ are constants and that the shape of the planet is a sphere, or at least a spheroid of rotation about the polar axis that bulges outward at the Equator.

None of these simplifications is fully accurate. Nevertheless, a common theoretical practice is to act as if they are true, with the proviso that the fluid dynamical equations are fundamentally expressed in geopotential coordinates, with the vertical direction $\hat{{\mathbf {z}}}$ defined as parallel to the spatial gradient of the combined gravitational-rotational potential, $-\, \nabla \Phi$, and with orthogonal horizontal coordinates, e.g., in the directions of longitude and latitude^1^. The advantages of this practice are to hide the complexity of the true gravity and rotation in a simple coordinate frame and, in particular, to be able to express the widespread approximate hydrostatic force balance between pressure gradient and density in only the vertical direction.

Chu^2^ asserts that the scientific community has hitherto neglected a potentially important, “horizontal” component of Earth’s gravitational acceleration. This is a misrepresentation of the community-standard formulation of the equations of Geophysical Fluid Dynamics (GFD), discussed in the preceding paragraph. The author claims instead that the widespread practice is to describe spatial variations on Earth in an absolute spherical coordinate system. In such a coordinate system the elevations of geopotential surfaces vary as functions of latitude and longitude, and thus there is a horizontal component of the gravitational acceleration. The author asserts that this component of gravity has simply been neglected in previous studies of the ocean and atmosphere. In fact, the implicit use of geopotential coordinates in the standard formulation of the equations of GFD ensures that there is no horizontal component of gravity.

Below we provide a brief overview of the history of geopotential coordinates in GFD, to demonstrate the extent to which Chu^2^ misrepresents this standard practice. We then explicitly consider the formulation of the equations describing ocean circulation in an absolute spherical coordinate system, as suggested by Chu^2^. We show that even in such a coordinate system Chu^2^ vastly overestimates the importance of the “horizontal gravity” terms. Finally, we show that subsequently published findings by Chu^3,4^, built upon the “horizontal gravity” formulation of Chu^2^, erroneously attribute modifications of the flow in oceanic and atmospheric Ekman layers to the effects of “horizontal gravity”.

## Historical use of geopotential coordinates to describe geophysical flows

The practice of using geopotential coordinates in Geophysical Fluid Dynamics has deep roots. It may have arisen in the context of Earth’s tides, and it is certainly implicit in the Laplace tidal equation^5^ (likely with overlap with work by Lagrange^6,7^; see review by Craik^8^). The predictions of these equations have been validated extensively against observations^9^. At least by the time of Lamb^10^ (Sec. 213), it was recognized that the appropriate coordinate frame was relative to a resting free surface under the influence of both gravity and Earth’s rotation (i.e. centrifugal force), which we can equate to a “geopotential” surface, $\Phi =$ constant. Explicitly he says “denote by *z* the altitude measured outwards along a normal, of any point on this surface”, i.e. in the direction of $-\,\nabla \Phi$. He also declared that this surface “is a surface of revolution about the polar axis, but the ellipticity will not in the first instance be taken to be small”. We cannot discern whether Lamb recognized that this ellipsoid was partly due to the non-uniformity of the gravity field, as well as to the centrifugal force. We believe there was no observational evidence about the geographical variation of ${\vec {g}} = -\, \nabla \Phi$ at that time.

The tides are more generally discussed by Hendershott and Munk^9^ and by Hendershott^11^. In the latter is a statement that “it is convenient to represent the tide-generating potential by its horizontal and time variation over some near-sea-level equipotential (the geoid) of the gravitational potential due to the earth’s shape, internal mass distribution, and rotation”. Obviously, the variation of ${\mathbf {g}}$ was known by then. Modern measurements^1^ have shown that the radius of an iso-potential surface near Earth’s sea surface varies by ${\mathcal {O}}(100)$ m, which is small compared to the mean radius of $\approx$ 6368 km.

Phillips^12^ writes equations for large-scale flows as a template for modern global atmospheric and oceanic models that explicitly implicate $-\,\nabla \Phi$ as the direction of the vertical coordinate and he refers to $\Phi$ as the geoid that “is not exactly a spherical surface of revolution but undulates slightly above and below the reference spheroid. The latter is a theoretically and empirically defined ellipsoid of revolution whose shape and mass is such that [it] ...gives a good approximation to observed gravity.” He also identifies a geographic latitude as the angle between ${\mathbf {g}}$ and the equatorial plane, with longitude the third orthogonal coordinate direction. He even discusses a formal curvilinear coordinate representation in terms of the reference spheroid, though he soon shifts to spherical coordinates.

Therefore, the basis for preferring geopotential coordinates, in which the gravitational force acts vertically, is well established in GFD. In this coordinate system gravity acts only in the vertical direction, so there is no “horizontal gravity” missing from previous GFD studies. Chu^2^ presents equations of motion in an alternative, absolute spherical coordinate system, in which the elevations of geopotentials vary with latitude and longitude, and thus there is a horizontal component of the gravitational acceleration. However, this formulation is much less convenient for oceanic or atmospheric models due to the large (${\mathcal {O}}(100\,{\mathrm {m}}))$ undulations of the ocean surface associated with the marine geoid.

## Negligible “horizontal gravity” in an absolute spherical coordinate system

Chu^2^ derives a formulation of the Boussinesq equations of motion in an absolute spherical coordinate system. They compare the “horizontal gravity” terms that emerge in this coordinate system with the magnitude of the Coriolis force at the ocean surface, as derived from Ocean Surface Current Analysis Real-time (OSCAR) surface currents. The Coriolis force is particularly relevant because its balance with the horizontal gradient of the sea surface elevation (relative to the geoid) determines the horizontal, geostrophic surface current. The author finds that the horizontal gravity force is typically an order of magnitude larger than the Coriolis force. The author concludes that existing formulations of ocean dynamics are missing a key contribution to the horizontal momentum balance because they do not account for “horizontal gravity”.

As noted above, existing formulations of ocean dynamics are, in fact, posed in geopotential coordinates, so there is no “horizontal gravity” force that needs to be accounted for. If one insists on formulating the equations of motion in an absolute spherical coordinate system then “horizontal gravity” terms do indeed appear. However, below we show that the comparison performed by Chu^2^ grossly overestimates the importance of this “horizontal gravity” force. This is because the “horizontal gravity” force is largely compensated by the horizontal component of the hydrostatic pressure, which reduces its effective strength by three orders of magnitude.

The horizontal momentum equation (Eq. (22) of Chu^2^) is

1 $$\begin{aligned} \underbrace{\rho _0\frac{{\mathrm {D}} \varvec{U}}{{\mathrm {D}} t}}_{\text {Advection}} \quad +\quad \underbrace{\rho _0 f\hat{\varvec{z}} \times \varvec{U}}_{\text {Coriolis}} \quad +\quad \underbrace{\nabla _h p}_{\text {Pressure gradient}} = \underbrace{\rho \nabla _h V}_{\text {Horizontal gravity}} + \underbrace{\rho _0 \varvec{F},}_{\text {External forcing}} \end{aligned}$$

and the vertical momentum equation (Eq. (26) of Chu^2^) is

2 $$\begin{aligned} \frac{\partial p}{\partial z} = \rho \frac{\partial V}{\partial z} {.}\end{aligned}$$

Here $\rho _0$ is the reference density, $\rho$ is the spatially-varying density, *f* is the Coriolis parameter, $\varvec{U}$ is the horizontal velocity vector, *p* is the pressure, $\nabla _h$ denotes the horizontal gradient operator, and $\varvec{F}$ denotes additional sources and sinks of momentum. Here it is assumed that the tilt of geopotential surfaces relative to spheroidal surfaces is small, such that the conventional assumption of a small vertical-to-horizontal aspect ratio holds^13^. The gravitational *V* is given approximately by (Eq. (16) of Chu^2^)

3 $$\begin{aligned} V \approx g_0 (N - z) , \end{aligned}$$

where $g_0$ is the reference gravitational acceleration and *N* is the horizontally-varying elevation of a reference geopotential surface that everywhere lies below the sea surface.

Chu^2^ compares the “horizontal gravity” and Coriolis terms in (1), and finds the former to exceed the latter by an order of magnitude for typical ocean surface currents. However, this comparison overlooks an almost complete compensation between the “horizontal gravity” term and the horizontal pressure gradient term. Noting that $\partial V/\partial z \approx -g_0$ from (3), we integrate (2) downward from the sea surface ($z=S$) to obtain

4 $$\begin{aligned} p = - g_0 \int _{z^\prime = S}^{z^\prime = z} \rho \,{\mathrm {d}}z^\prime {.}\end{aligned}$$

We can then write the combined horizontal pressure gradient and “horizontal gravity” terms in (1) as

5 $$\begin{aligned}&  \rho _0\frac{{\mathrm {D} }\varvec{U}}{{\mathrm {D}} t} + \rho _0 f\hat{\varvec{z}} \times \varvec{U} - \rho _0 \varvec{F} = - \nabla _h p + \rho \nabla V = g_0 \int _{z^\prime = S}^{z^\prime = z} \nabla _h \rho \,{\mathrm {d}}z^\prime - \rho _0 g_0 \nabla _h S + g_0 \rho \nabla _h N \\&\quad  = \underbrace{g_0 \int _{z^\prime = S}^{z^\prime = z} \nabla _h \rho \,{\mathrm {d}}z^\prime }_{\text {Baroclinic pressure gradient}} \underbrace{-\rho _0 g_0 \nabla _h (S-N)}_{\text {Surface pressure gradient}} +\quad \underbrace{g_0 (\rho -\rho _0) \nabla _h N}_{\text {Horizontal gravity anomaly}} , \end{aligned}$$

where $\rho _0 = \rho |_{z=S}$ is the surface density. In Eq. (5), the surface pressure gradient is now expressed in terms of the surface relative to the geopotential surface. Eq. (5) is identical to the expression for the horizontal pressure gradient that would be obtained in conventional geopotential coordinates^14^, plus an additional “horizontal gravity anomaly” term. Thus the appropriate comparison to assess the importance of “horizontal gravity” in this formulation of the equations of motion is to compare the “horizontal gravity anomaly” and Coriolis terms in (5). At the surface $z=S$ the “horizontal gravity anomaly” term is zero by construction because $\rho =\rho _0$. In the subsurface, while the “horizontal gravity anomaly” term in (5) is non-zero, it is approximately three orders of magnitude smaller than the “horizontal gravity” term in (1). Based on the finding of Chu^2^ that the “horizontal gravity” term is an order of magnitude larger than the Coriolis force, it is reasonable to expect that the “horizontal gravity anomaly” is typically one to two orders of magnitude smaller than the Coriolis force.

In summary, contrary to Chu’s claims^2^, the conventional formulation of ocean dynamical equations is posed implicitly in geopotential coordinates. In this coordinate system there is no horizontal component of the gravitational force, and thus “horizontal gravity” does not exist. If one were to formulate the equations of motion in an absolute spherical coordinate system, with spatially-varying geopotential elevations, then these equations should indeed include a term due to “horizontal gravity” (c.f. Eq. 1). However the effect of this term is almost completely compensated by the horizontal gradient of the hydrostatic pressure, with the residual “horizontal gravity anomaly” term being typically three orders of magnitude smaller. Consequently, “horizontal gravity” would likely have a negligible impact on ocean circulation even in a model formulated in absolute spherical coordinates.

## “Horizontal gravity” does not modify the oceanic nor atmospheric Ekman layers

This section discusses findings reported in publications by Chu^3,4^, which build directly upon the work of Chu^2^. The articles examine the influence of “horizontal gravity” on flows in the oceanic and atmospheric Ekman layers. As noted above, existing formulations of ocean dynamics are, in fact, posed in geopotential coordinates, so there is no “horizontal gravity” force that needs to be accounted for in these Ekman layers. However, if one insists on formulating the equations of motion in an absolute spherical coordinate system then “horizontal gravity” terms do indeed appear.

In both articles the author concludes that “horizontal gravity” dramatically alters the flow in the Ekman layer, and thus should be accounted for in models of the ocean and atmosphere. This would be a remarkable result, given that it results from only a slight change in the coordinate system (from geopotential coordinates to absolute spherical coordinates). However, it is misleading to include “horizontal gravity” in the momentum balance of the Ekman layer. Instead, “horizontal gravity” and the horizontal pressure gradient jointly define both the resting hydrostatic state and the geostrophic flow that the Ekman layer solution should approach far from the atmosphere-ocean interface. This adjustment leaves the classical Ekman layer solution unmodified by the switch from geopotential to absolute spherical coordinates. This argument is explained in more detail in the Appendix.

In summary, contrary to the claims of Chu^3^ and Chu^4^, the conventional formulation of GFD in geopotential coordinates eliminates any horizontal component of the gravitational force. Thus, under this formulation “horizontal gravity” does not exist and has no impact on the flow in the oceanic nor atmospheric Ekman layers. If one formulates the equations of motion in an absolute spherical coordinate system, with spatially-varying geopotential elevations, then a term due to “horizontal gravity” does appear. However, it is misleading to label the flows resulting from this force as a component of the Ekman flow; rather, the combination of the horizontal pressure gradient and “horizontal gravity” forces define the hydrostatic resting state and the geostrophic flow outside the Ekman layer, and the ageostrophic Ekman transport should be defined as the deviation from this geostrophic flow. Under this decomposition the Ekman flow in absolute spherical coordinates is identical to the Ekman flow in conventional geopotential coordinates.

## Discussion

In summary, we find Chu’s advocacy of horizontal gravity to be erroneously presented and, more importantly, inapposite even if correctly done. The foundational ideas of geostrophic, hydrostatic balance and Ekman boundary layers remain intact. The value of satellite altimetry for estimating large- and meso-scale oceanic surface geostrophic currents is preserved (to paraphrase Walter Munk, “the greatest experiment ever done in physical oceanography”).

## Supplementary Information

Supplementary Information.
