# Supplementary material for: Gravity is vertical in Geophysical Fluid Dynamics
Source: Sci Rep. 2022 Apr 11;12:6029. doi: 10.1038/s41598-022-10023-3 (PMC9001706; doi:10.1038/s41598-022-10023-3)
Supplement: Supplementary file 1 — Supplementary Information. [file 41598_2022_10023_MOESM1_ESM.pdf]

Supplementary Information for ‘Gravity is Vertical in Geophysical Fluid Dynamics’

Andrew L. Stewart[1,*] **and James C. McWilliams**[1]

[1]Department of Atmospheric and Oceanic Sciences, University of California, Los Angeles, California, USA
[*]astewart@atmos.ucla.edu

Appendix

“Horizontal gravity” does not modify oceanic Ekman transport

The analysis of Chu[1] starts from the Boussinesq momentum equation, posed in an absolute spherical coordinate system in the limit of small Rossby number (Eq. 17 of Chu[1]):

$$\underbrace{\rho_0 f \hat{\boldsymbol{z}} \times \boldsymbol{U}}_{\text{Coriolis}} + \underbrace{\nabla_3 p}_{\text{Pressure gradient}} \approx \underbrace{\rho \boldsymbol{g}}_{\text{Gravity}} + \underbrace{\rho_0 \boldsymbol{F}}_{\text{External forcing}} . \tag{1}$$

Here ρ_0 is the reference density, ρ is the spatially-varying density, f is the Coriolis parameter, $\boldsymbol{U}$ is the horizontal velocity vector, p is the pressure, ∇_3 denotes the three-dimensional gradient operator, and $\boldsymbol{F}$ denotes additional sources and sinks of momentum. Note that the gravity vector $\boldsymbol{g}$ has both horizontal and vertical components, $\boldsymbol{g} = \boldsymbol{g}_h + g_z \hat{\boldsymbol{z}}$. Following Chu[1], we write these as

$$\boldsymbol{g}_h \approx g_0 \nabla_h N, \tag{2a}$$

$$g_z = -g_0, \tag{2b}$$

where N is the elevation of the marine geoid, g_0 is the reference gravitational acceleration, and ∇_h denotes the horizontal gradient operator.

The central issue with the analysis of Chu[1] lies in the identification of a “geostrophic” component that balances the horizontal pressure gradient via the Coriolis force:

$$\rho_0 f \hat{\boldsymbol{z}} \times \boldsymbol{U}_g^{\mathrm{C21}} = -\nabla_h p. \tag{3}$$

The remaining component of the flow balances the horizontal component of the gravity vector and the external forcing via the Coriolis force, and is referred to as the “ageostrophic” or “Ekman” flow:

$$\rho_0 f \hat{\boldsymbol{z}} \times \boldsymbol{U}_E^{\mathrm{C21}} = \rho \boldsymbol{g}_h + \rho_0 \boldsymbol{F}. \tag{4}$$

Here the superscript $^{\mathrm{C21}}$ denotes definitions made by Chu[1]. We argue that this is an inappropriate decomposition, and that in fact the horizontal component of gravity should be included in the “geostrophic” flow, rather than the “ageostrophic”/“Ekman” flow. The basis for this argument is that: (i) the horizontal component of the gravity vector is almost entirely compensated by the horizontal component of the hydrostatic pressure, so this separation produces components of the flow that largely cancel one another; (ii) the residual between the horizontal pressure gradient force and horizontal gravity produces horizontal flows throughout the water column, rather than just in the surface boundary layer; (iii) the formulation of Chu[1] implies that even in the absence of wind forcing $\boldsymbol{F} = 0$, there is still an ageostrophic Ekman transport driven by the “horizontal gravity”, which contradicts established theory and measurements of the wind-driven ocean surface boundary layer[2]. Thus, both the horizontal pressure gradient and the “horizontal gravity” jointly determine the geostrophic flow external to the Ekman layer.

In support of this argument, first write the density as a perturbation relative to the constant reference density,

$$\rho = \rho_0 + \tilde{\rho}. \tag{5}$$

Next, note that the gravity term in (1) corresponding to the reference density ρ_0 can be written as an exact gradient,

$$\rho \boldsymbol{g} = \rho_0 \boldsymbol{g} + \tilde{\rho} \boldsymbol{g} = \nabla_3 \Phi_0 + \tilde{\rho} \boldsymbol{g}, \tag{6}$$

where the reference gravitational potential is given by

$$\Phi_0 = \rho_0 g_0 (N - z). \tag{7}$$

We can therefore define a reference hydrostatic pressure p_0 that exactly balances the component of gravity associated with the reference density, *i.e.*

$$p = p_0 + \tilde{p}, \qquad \nabla_3 p_0 = \nabla_3 \Phi_0. \tag{8}$$

Substituting (8) into the horizontal component of (1) yields

$$\underbrace{\rho_0 f \hat{\boldsymbol{z}} \times \boldsymbol{U}}_{\text{Coriolis}} \approx \underbrace{-\nabla_h \tilde{p}}_{\text{Pressure gradient}} + \underbrace{\tilde{\rho} g_0 \nabla_h N}_{\text{Horizontal gravity}} + \underbrace{\rho_0 \boldsymbol{F}}_{\text{External forcing}} . \tag{9}$$

This form of the horizontal momentum equation largely removes the issue of compensation between the horizontal pressure gradient force and the horizontal gravity force, which would otherwise greatly exaggerate the dynamical influence of the "horizontal gravity term".

We now consider the horizontal flows produced by the action of each of the terms on the right-hand side of (9). Following Chu[1], we consider an external forcing $\boldsymbol{F}$ resulting from vertical eddy momentum fluxes,

$$\boldsymbol{F} = \frac{\partial}{\partial z}\left(K \frac{\partial \boldsymbol{U}}{\partial z}\right), \tag{10}$$

where K is the eddy viscosity. The surface boundary condition is

$$\rho_0 K \frac{\partial \boldsymbol{U}}{\partial z} = \boldsymbol{\tau} \qquad \text{at} \qquad z = 0, \tag{11}$$

where $\boldsymbol{\tau}$ is the surface wind stress, and we assume that the eddy viscosity becomes negligibly small outside the surface boundary layer,

$$K \to 0 \qquad \text{for} \qquad z < -H_{\text{bl}}, \tag{12}$$

where H_{bl} denotes the thickness of the surface boundary layer. By integrating (9) from the surface to an arbitrary elevation $z < -H_{\text{bl}}$, we can then write an expression for the transport above that elevation:

$$\underbrace{\int_z^0 \boldsymbol{U}\, \mathrm{d}z'}_{\text{Total transport}} = \underbrace{\frac{1}{\rho_0 f}\int_z^0 \hat{\boldsymbol{z}} \times \nabla_h \tilde{p}\, \mathrm{d}z'}_{\text{Pressure gradient}} + \underbrace{\frac{1}{f}\int_z^0 b \hat{\boldsymbol{z}} \times \nabla_h N\, \mathrm{d}z'}_{\text{Horizontal gravity}} \underbrace{- \frac{1}{\rho_0 f} \hat{\boldsymbol{z}} \times \boldsymbol{\tau}}_{\text{Ekman transport}} . \tag{13}$$

Here $b = -g\tilde{\rho}/\rho_0$ is the buoyancy. The last term in (13) is the familiar expression for the Ekman transport[3]; this component of the transport is confined entirely to the surface boundary layer. In contrast, both the pressure gradient and "horizontal gravity" components of the transport in (13) vary, in general, throughout the water column. Thus the pressure gradient and "horizontal gravity" set the far-field flow, below the Ekman layer, and the external forcing term in (9) drives the ageostrophic, Ekman layer flow.

Furthermore, arbitrary changes in the "horizontal gravity" term may be imposed simply by changing the reference density ρ_0. To be precise, consider a change in the reference density from ρ_0 to $\rho_0^\star = \rho_0 + \delta\rho_0$, where $\delta\rho_0 \ll \rho_0$ and $(\rho - \rho_0) \ll \rho_0$. The buoyancy becomes

$$b^\star = -g(\rho - \rho_0^\star)/\rho_0^\star \approx b + \delta b_0, \qquad \delta b_0 = g\delta\rho_0/\rho_0^\star, \tag{14}$$

where we have approximated $\rho_0^\star \approx \rho_0$ in the denominator. Thus (13) becomes, after some manipulations,

$$\underbrace{\int_z^0 \boldsymbol{U}\, \mathrm{d}z'}_{\text{Total transport}} \approx \underbrace{\frac{1}{\rho_0 f}\int_z^0 \hat{\boldsymbol{z}} \times \nabla_h \tilde{p}^\star\, \mathrm{d}z'}_{\text{Pressure gradient}} + \underbrace{\frac{1}{f}\int_z^0 b \hat{\boldsymbol{z}} \times \nabla_h N\, \mathrm{d}z' - z\frac{\delta b_0}{f} \hat{\boldsymbol{z}} \times \nabla_h N}_{\text{Horizontal gravity}} \underbrace{- \frac{1}{\rho_0 f} \hat{\boldsymbol{z}} \times \boldsymbol{\tau}}_{\text{Ekman transport}} . \tag{15}$$

Here $\tilde{p}^\star$ is the perturbation pressure, defined relative to a reference pressure $p_0^\star$ that hydrostatically balances the reference gravitational potential $\Phi_0^\star = \rho_0^\star g_0 (N - z)$ (*c.f.* Eq. (8)). Eq. (15) implies that an arbitrary change in the reference density leads to a vertically-uniform addition to the "horizontal gravity"-driven component of the flow, and thus a vertically-integrated transport that increases linearly with depth. This implies that the "horizontal gravity"-driven component of the flow is ill-defined, and thus that analyzing this flow in isolation, or as part of the 'Ekman' transport (as done by Chu[1]) is misleading.

To reconcile the apparent arbitrariness in the definition of the "horizontal gravity"-driven component of the flow in Eq. (15), we note that the perturbation pressure gradient may be written, using (8), as

$$\nabla_h \tilde{p} = \nabla_h \tilde{p}^{\star} + \delta\rho_0 g_0 \nabla_h N. \tag{16}$$

Substituting Eq. (16) into Eq. (15) we obtain, after some manipulations,

$$\underbrace{\int_z^0 \boldsymbol{U}\,\mathrm{d}z'}_{\text{Total transport}} \approx \underbrace{\frac{1}{\rho_0 f}\int_z^0 \hat{\boldsymbol{z}}\times\nabla_h\tilde{p}\,\mathrm{d}z' + z\frac{\delta b_0}{f}\hat{\boldsymbol{z}}\times\nabla_h N}_{\text{Pressure gradient}} + \underbrace{\frac{1}{f}\int_z^0 b\hat{\boldsymbol{z}}\times\nabla_h N\,\mathrm{d}z' - z\frac{\delta b_0}{f}\hat{\boldsymbol{z}}\times\nabla_h N}_{\text{Horizontal gravity}} \underbrace{-\frac{1}{\rho_0 f}\hat{\boldsymbol{z}}\times\boldsymbol{\tau}}_{\text{Ekman transport}}. \tag{17}$$

Thus the shift in the reference density produces equal and opposite perturbations to the pressure gradient-driven and "horizontal gravity"-driven components of the flow. Simplifying Eq. (17) by cancelling these terms recovers Eq. (13). This highlights the close dynamical link between the pressure gradient and "horizontal gravity" terms, and indicates that they should be considered to set the geostrophic component of the flow in tandem.

Based on these considerations, we argue that in an absolute spherical coordinate system, the appropriate decomposition of the flow would be to relate the geostrophic flow to the residual between the horizontal pressure gradient and "horizontal gravity",

$$\rho_0 f\hat{\boldsymbol{z}}\times\boldsymbol{U}_g = -\nabla_h p + \rho\boldsymbol{g}_h. \tag{18}$$

It follows that the "ageostrophic" or "Ekman" flow is

$$\rho_0 f\hat{\boldsymbol{z}}\times\boldsymbol{U}_E = \rho_0\boldsymbol{F}. \tag{19}$$

Thus the "Ekman" flow is unchanged by the shift to absolute spherical coordinates.

"Horizontal gravity" does not modify atmospheric Ekman pumping

The analysis of Chu[4] starts from the same momentum equation as that of Chu[1] (Eq. 19a of Chu[4]), but now describes flow in the atmosphere:

$$\underbrace{\rho_0 f\hat{\boldsymbol{z}}\times\boldsymbol{U}}_{\text{Coriolis}} + \underbrace{\nabla_3 p}_{\text{Pressure gradient}} \approx \underbrace{\rho\boldsymbol{g}}_{\text{Gravity}} + \underbrace{\rho_0\boldsymbol{F}}_{\text{External forcing}}. \tag{20}$$

Here ρ_0 is the reference density, ρ is the spatially-varying density, f is the Coriolis parameter, $\boldsymbol{U}$ is the horizontal velocity vector, p is the pressure, ∇_3 denotes the three-dimensional gradient operator, and $\boldsymbol{F}$ denotes additional sources and sinks of momentum. Note that the gravity vector $\boldsymbol{g}$ has both horizontal and vertical components, $\boldsymbol{g} = \boldsymbol{g}_h + g_z\hat{\boldsymbol{z}}$. The analysis of Chu[4] proceeds by identifying a "geostrophic" component of the flow that balances the horizontal pressure gradient via the Coriolis force:

$$\rho_0 f\hat{\boldsymbol{z}}\times\boldsymbol{U}_g^{\mathrm{C21}} = -\nabla_h p. \tag{21}$$

The remaining component of the flow balances the horizontal component of the gravity vector and the external forcing via the Coriolis force, and is referred to as the "ageostrophic" or "Ekman" flow:

$$\rho_0 f\hat{\boldsymbol{z}}\times\left(\boldsymbol{U}-\boldsymbol{U}_g^{\mathrm{C21}}\right) = \rho\boldsymbol{g}_h + \rho_0\boldsymbol{F}. \tag{22}$$

Here the superscript $^{\mathrm{C21}}$ denotes definitions made by[1]. The author then solves (22) for the vertical structure of the "ageostrophic" flow, $\left(\boldsymbol{U}-\boldsymbol{U}_g^{\mathrm{C21}}\right)$, throughout the atmospheric boundary layer, under the canonical assumption that the external forcing $\boldsymbol{F}$ takes the form of a vertical Laplacian operator with a constant eddy viscosity[3].

This decomposition, from which all of the analysis of Chu[4] is derived, is critically flawed. The "horizontal gravity" force, which appears in (20) only because an absolute spherical coordinate system is used, should contribute to the definition of the geostrophic flow, rather than the ageostrophic/Ekman flow. Far above the atmospheric Ekman layer, *i.e.* for $z \gg D_E$, the eddy vertical viscosity may be neglected and thus $\boldsymbol{F} = 0$. Thus the far-field horizontal velocity is given by

$$\rho_0 f\hat{\boldsymbol{z}}\times\boldsymbol{U} = -\nabla_h p + \rho\boldsymbol{g}_h \quad \text{for} \quad z \gg D_E. \tag{23}$$

The canoncial formulation of the atmospheric Ekman layer[3] describes the behavior of a viscous boundary layer in response to an externally-imposed, geostrophic flow. In the absolute spherical coordinate system used by Chu[4] the externally-imposed flow is given by (23). Therefore, in this coordinate system the appropriate decomposition would be to define the geostrophic flow as

$$\rho_0 f \hat{\boldsymbol{z}} \times \boldsymbol{U}_g = -\nabla_h p + \rho \boldsymbol{g}_h, \tag{24}$$

and the "ageostrophic" or "Ekman" flow as

$$\rho_0 f \hat{\boldsymbol{z}} \times \left(\boldsymbol{U} - \boldsymbol{U}_g\right) = \rho_0 \boldsymbol{F}. \tag{25}$$

Thus the "Ekman" flow and pumping are unchanged by the shift to absolute spherical coordinates.

References

1. Chu, P. C. True gravity in ocean dynamics. Part 1: Ekman transport. *Dyn. Atmospheres Ocean.* **96**, 101268 (2021).

2. Svensson, U. The structure of the turbulent Ekman layer. *Tellus* **31**, 340–350 (1979).

3. Pedlosky, J. *Geophysical fluid dynamics* (Springer Science & Business Media, 2013).

4. Chu, P. C. True gravity in atmospheric ekman layer dynamics. *J. Geophys. Res. Atmospheres* **126**, e2021JD035293 (2021).
